# CHRONIC CONDITIONS, FUNCTIONAL LIMITATIONS, AND DEPRESSION IN OLDER ADULTS: ANALYSIS OF A LONGITUDINAL STUDY

**DOI:** 10.1093/geroni/igz038.2118

**Published:** 2019-11-08

**Authors:** Jyotsana Parajuli, Diane Berish, Ying-Ling Jao

**Affiliations:** 1 The Pennsylvania State University, College of Nursing, State College, Pennsylvania, United States; 2 Pennsylvania State University, University Park, Pennsylvania, United States

## Abstract

Background: Chronic conditions, functional limitations, and depression are highly prevalent in older adults. Evidence suggests the links between chronic conditions, functional limitations, and depressive symptoms separately. However, few studies have considered these three conditions together longitudinally. This study examined the longitudinal relationship between chronic conditions and depressive symptoms and evaluated the mediation effect of functional limitations on the relationship between chronic conditions and depressive symptoms in older adults. Methods: This study analyzed longitudinal data from the Health and Retirement Study collected in 2012 and 2014. Mediation analysis was used to examine the direct and indirect effects of chronic conditions and functional limitations measured at the year 2012 on depressive symptoms measured at the year 2014 controlling for demographics. Results: Results revealed that chronic conditions predicted depressive symptoms. Specifically, one additional chronic condition in 2012 corresponded to an increase of 0.35 in depressive symptoms in 2014 (p<.001). After adding functional limitations as a mediator, the direct effect was reduced to 0.26 and the indirect effect was .088 (p<.001). In other words, functional limitations explained approximately 25% of the direct effect of chronic diseases on depression. Discussion: Findings reveal the longitudinal impact of chronic conditions and functional limitations on depressive symptoms in older adults. Findings help identify the high-risk population of depressive symptoms and intervene early.

